# Erythromycin Treatment of *Brassica campestris* Seedlings Impacts the Photosynthetic and Protein Synthesis Pathways

**DOI:** 10.3390/life10120311

**Published:** 2020-11-26

**Authors:** Young-Eun Yoon, Hyun Min Cho, Dong-won Bae, Sung Joong Lee, Hyeonji Choe, Min Chul Kim, Mi Sun Cheong, Yong Bok Lee

**Affiliations:** 1 Division of Applied Life Science (BK21four), Gyeongsang National University, Jinju-daero 501, Jinju 52665, Korea; yye209@gnu.ac.kr (Y.-E.Y.); hmcho86@gnu.ac.kr (H.M.C.); mulberry1028@gnu.ac.kr (H.C.); mckim@gnu.ac.kr (M.C.K.); 2 Center for Research Facilities, Gyeongsang National University, Jinju-daero 501, Jinju 52665, Korea; bdwon@gnu.ac.kr; 3 Institute of Agriculture & Life Science, Gyeongsang National University, Jinju-daero 501, Jinju 52665, Korea; mtws@nate.com

**Keywords:** antibiotics, erythromycin (Ery), *Brassica campestris*, proteomics, photosynthesis, protein synthesis, ribosome

## Abstract

Erythromycin (Ery) is a commonly used veterinary drug that prevents infections and promotes the growth of farm animals. Ery is often detected in agricultural fields due to the effects of manure application in the ecosystem. However, there is a lack of information on Ery toxicity in crops. In this study, we performed a comparative proteomic analysis to identify the molecular mechanisms of Ery toxicity during seedling growth based on our observation of a decrease in chlorophyll (Chl) contents using *Brassica campestris*. A total of 452 differentially abundant proteins (DAPs) were identified including a ribulose-1,5-bisphosphate carboxylase (RuBisCO). The proteomic analysis according to gene ontology (GO) classification revealed that many of these DAPs responding to Ery treatment functioned in a cellular process and a metabolic process. The molecular function analysis showed that DAPs classified within catalytic activity were predominantly changed by Ery, including metabolite interconversion enzyme and protein modifying enzyme. An analysis of functional pathways using MapMan revealed that many photosynthesis components were downregulated, whereas many protein biosynthesis components were upregulated. A good relationship was observed between protein and transcript abundance in a photosynthetic pathway, as determined by qPCR analysis. These combined results suggest that Ery affects plant physiological activity by downregulating protein abundance in the photosynthetic pathway.

## 1. Introduction

Antibiotics have been used as chemotherapeutic agents in human and veterinary medicine for many decades, and the use of three biologically active molecules has increased globally [1,2]. These substances affect agricultural environments due to human behaviors such as soil fertilization during composting of sludge or manure [3,4,5]. These contaminants accumulate in the soil and affect the soil ecosystem and microbial communities according to their antibiotic resistance [6,7].

Agricultural crops are exquisitely sensitive to their external environments; climate conditions and soil nutrients affect plant growth and development as well as crop yield and quality [8,9]. *Brassica campestris*, also known as Kimchi cabbage, is a nutritionally rich vegetable that is cultivated worldwide [10]. The early growth of cabbage seedlings is important for forming the leafy head, which affects crop quality [11]. Modern intensive and short-term monoculture requires high inputs of fertilizer and chemicals; thus, composting is a common method to improve soil nutrient contents [12].

Veterinary drugs such as antibiotics are used for disease treatment or prevention, and the use of antibiotics for animal husbandry is at least five times higher than that for humans [3]. Approximately 30–90% of active antibiotic intake is excreted due to low absorption rate in the body [13]. Excreted antibiotics remain in manure during the process of composting and are subsequently released into the agricultural field [3,4,5]. Antibiotics are an environmental factor affecting plant growth and development including woody plants such as *Populus alba* [14,15]; however, their effects on crop cultivation are largely unknown.

Plant leaves are the major organs of photosynthesis, and leaf color is significantly related to photosynthesis efficiency and influence in plant growth and development such as senescence [16]. Light-driven photosynthetic reactions in the thylakoid membrane include not only the photon-triggered electron transport chain in photosystem II (PSII), the cytochrome b6f complex, photosystem I (PSI) but also the free electron carriers such as plastoquinone and plastocyanin [17,18]. The two light reactions work sequentially, where electrons extracted from water in PSII are transferred through the plastoquinone pool (PQ), the cytochrome b6f complex (Cyt), and plastocyanin (PC) to PSI, and ultimately to ferredoxin and NADP^+^ to produce NADPH [18]. These electron transfer reactions are coupled with proton pumping into the thylakoid lumen, and the resulting proton gradient is utilized to generate adenosine triphosphate (ATP). ATP and NADPH fuel the Calvin cycle for CO_2_ fixation and assimilation [17].

d-ribulose-1,5-bisphosphate carboxylase/oxygenase (RuBisCO) catalyzes CO_2_ fixation in the photosynthetic carbon reduction (Calvin) cycle by combining CO_2_ with ribulose-1,5-bisphosphate (RuBP) to produce two 3-phosphoglycerate (3-PGA) [19]. RuBisCO is a ubiquitous protein located in the chloroplast stroma and is considered to be the most abundant plant protein by accounting for ~3% of the total leaf mass dry weight [20].

The functional components in the chloroplast are coordinately regulated in both the nucleus and chloroplast for protein expression and subunit assembly [21,22]. Some photosynthetic subunits encoded in the chloroplast genome are synthesized on chloroplastic ribosomes, and others encoded by nuclear genes are translated in the cytoplasm and imported into the chloroplast for assembly into functional complexes [18,21]. In addition, photosynthesis efficiency are regulated by environmental conditions such as light quality, CO_2_ level, temperature, and nutrients [23], thus suggesting that the activity of photosynthetic apparatus is affected by environmental factors, which influence gene expression and protein translation and modification [24].

Proteomic analysis and gene ontology (GO) classification are powerful tools for comprehensive examination of molecular mechanisms involved in specific biological processes, subcellular organelles, and functional protein pathways [25]. Proteins directly participate in plant physiological phenotypes. Protein function depends on molecular structure and subcellular localization (e.g., chloroplasts and mitochondria), because different cell organelles provide different physiological and biochemical environments [26].

Erythromycin (Ery) is one of the major antibiotic macrolides, which are large-spectrum antibiotics with bacteriostatic activity [27]. Ery inhibits protein synthesis by binding the 50S ribosomal subunit [28]. In this study, we investigated Ery toxicity on *B. campestris* seedlings by performing a comparative proteomic analysis. We identified numerous differentially abundant proteins (DAPs) in response to Ery, which were predominantly classified in the photosynthesis pathway and protein biosynthesis pathway. These results provide novel insights into the metabolic and physiological plant responses to Ery antibiotics and suggest that environmental antibiotics can adversely affect crop plant growth and development.

## 2. Materials and Methods

### 2.1. Erythromycin Treatment and Measurement of Plant Growth and Phyiological Parameters

Napa cabbage (*Brassica campestris* L. ssp. *perkinensis Rupr*) seeds were purchased from ASIA seed company (Seoul, Korea). To conduct the seedling growth assay, 36–50 seeds were placed on 1.2% agar media in the presence of Ery (TCI Development, Shanghai, China). Seeds were incubated vertically for 4–5 days in a growth chamber maintained at 22 °C under long-day photoperiod (16 h light/8 h dark) with 200 μEm^−2^S^−1^ light intensity using fluorescent lamps. Photographs of seedlings were captured, and primary root length was measured using ImageJ software (http://imagej.nih.gov/ij/download.html; Bethesda, MD, USA). Chlorophyll was extracted from detached cotyledons using methanol, and the contents of chlorophyll a (Chla) and chlorophyll b (Chlb) were calculated as follows: Chla = 16.72A_665.2_ − 9.16A_652.4_; Chlb = 34.09A_665.2_ − 15.28A_652.4_; A = absorbance [29].

### 2.2. Detection of Erythromycin Residue in Brassica campestris Seedlings

To detect Ery in *B. campestris* seedlings, shoots of 4-day-old seedlings grown in the presence of Ery (0, 5, and 10 mg/L) were harvested and washed three times with 50% methanol. To analyze accumulated Ery *in planta*, washed seedling tissues were freeze-dried, measured (dry weight), and milled using liquid nitrogen. Samples were prepared for liquid chromatography with tandem mass spectrometry (LC-MS/MS), as described Wang et al. [30].

### 2.3. Total Protein Extraction and One-Dimemnsional Gel Electrophoresis

For total protein extraction, harvested seedlings were ground into fine powder using liquid nitrogen, three volumes of ice-chilled protein extraction buffer [1 × PBS pH 7.4, 0.1% Triton X-100, protease inhibitor cocktail tablets (Complete Mini, Roche, Indianapolis, IN, USA)] were added, and the suspension was mixed well. The tubes were incubated in ice for 15 min and then centrifuged for 10 min at 4 °C. The supernatants (total protein extracts) were transferred to new tubes.

For one-dimensional SDS-PAGE, 50 μg of Napa cabbage (*Brassica campestris* subsp. *napus* var *pekinensis* MAKINO) total proteins were diluted with denaturing sample buffer (0.5 M Tris-HCl pH 6.8, 10% SDS, 20% glycerol, 1% bromophenol blue, 0.2% DTT) and heated at 95 °C for 5 min. Samples were subjected to SDS-PAGE, stained with Coomassie Brilliant R250 (Sigma-Aldrich, St. Louis, MO, USA), and then destained with water.

### 2.4. In-Gel Digestion

A one-dimensional SDS-PAGE lane containing all protein bands was excised from top to bottom using a razor blade, and excised gel slices were washed twice with 100 µL of distilled water for 15 min at room temperature. Excised gel bands were destained using acetonitrile. The gel slices were dried completely in a vacuum, and then alkylated by incubating with 55 mM iodoacetamide/0.1 M ammonium bicarbonate for 30 min at room temperature in the dark. After alkylation, the gel slices were dried again, and then the dried gel slices were swollen in digestion buffer (25 mM ammonium bicarbonate, 0.1% *n*-octyl glucoside, and 50 ng/mL of sequencing grade trypsin (Promega, Madison, MI, USA)) for rehydration. Peptides were extracted from the gel slices using 66% acetonitrile, 33% water, 0.1% trifluoroacetic acid (TFA). Extracted peptides were dried with a speedvac (Hanil, Korea), and stored at −80 °C before analysis [31].

### 2.5. LC-MS/MS Analysis

The dried peptides were redissolved in 20 µL of 5% formic acid and analyzed using on-line nanoflow LC-MS/MS. All nano-LC-MS/MS experiments were performed using an Ekisigent nanoLC415 system (EKsigent, Dublin, OH, USA) connected to Triple TOF 6600 mass spectrometry system (SCIEX, Redwood City, CA, USA) with a nanoelectron-spray ion source (New Objective, Woburn, MA, USA).

### 2.6. Data Analysis

After MS/MS analysis, data files were processed using UniProt and ProteinPilot 5.0.1 (SCIEX, Redwood City, CA, USA). Based on the combined MS and MS/MS spectra, proteins were successfully identified at ≥95% confidence interval using their scores in the MASCOT v 2.6 search engine (Matrix Science Ltd., London, UK) and the following search parameters: plant database (*Brassica campestris* subsp. *napus* var *pekinensis* MAKINO database), trypsin as the digestion enzyme, single missed cleavage sites, fixed carbamidomethyl (C) modifications and methionine oxidation, ±0.1 Da precursor ion tolerance, and ±0.1 Da MS/MS fragment ion tolerance. The database search results were manually curated to yield the protein identifications using 1% global false discovery rate (FDR) determined by the FDR tool in ProteinPilot software. Scaffold v 4.11.0 (Proteome Software Inc., Portland, OR, USA) was used to validate MS/MS-based peptide and protein identifications. The identified proteins were searched, and information on functional grouping was obtained using PANTHER (http://pantherdb.org) and STRING (http://string-db.org) databases for gene ontology (GO) analysis. MapMan software (v 3.6.0RC1; http://mapman.gabipd.org) was used for the functional pathway analysis [32]. A workflow chart of the proteomic analysis is presented in Figure S3.

### 2.7. RNA Extraction and qRT-PCR

Total RNA was extracted from *B. campestris* seedlings using TRIzol reagent (Thermo Fisher Scientific, Waltham, MA, USA) according to the manufacturer’s instructions, treated with DNase I (Thermo Fisher Sci, Waltham, MA, USA), and RNA was purified using a Riboclear Column (GeneAll, Seoul, Korea). First-strand cDNA was synthesized from 2 μg of total RNA using a cDNA synthesis kit (Thermo Fisher Scientific). Then, qRT-PCR was performed on a CFX Connect Real-Time PCR Detection System (Bio-Rad, Hercules, CA, USA) using the cDNA template, gene-specific primers (Supplementary Table S1), and AccuPower 2× GreenStar qPCR Master Mix (Bioneer, Daejeon, Korea). *EF1a* or *Act7* was used as the internal reference gene for data normalization. Average gene expression levels were determined using the comparative Ct method (2^−ΔΔCt^).

## 3. Results

### 3.1. Erythromycin Inhibits Brassia campestris Seedling Growth

**Figure 1:**
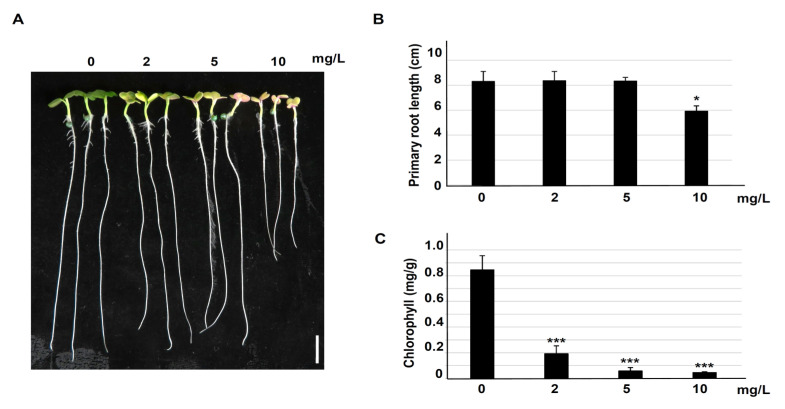
The effects of Ery on *Brassica campestris* seedling growth and development. Sterilized seeds were placed on Ery-containing agar medium and grown vertically for four days. (**A**) Morphological phenotype at day 4. (**B**) Primary root length. (**C**) Chlorophyll contents. Data represent mean ± SD (*n* = 48). All experiments were replicated four times with similar results. Asterisks indicate statistically significant difference from control (0 mg/L) (Student’s *t*-test; * *p* < 0.05, *** *p* < 0.001).

The effect of Ery on crop physiology, growth, and development was investigated in *B. campestris* seedlings (Figure 1). Seeds were placed on 1.2% agar media containing the indicated Ery concentration (0, 2, 5, and 10 mg/L), and the primary root lengths and chlorophyll contents were examined in cotyledons. High Ery concentration (10 mg/L) reduced primary root growth, whereas primary root growth was similar under low Ery concentrations (2 and 5 mg/L) and control conditions (0 mg/L Ery) (Figure 1B).

Primary root growth can be affected by radical emergence during seed germination. Therefore, we examined radicle emergence in the absence and presence of Ery (10 mg/L). Seed coat rupture was examined every 6 h after imbibition until radicle emergence. There was no significant difference between 0 and 10 mg/L Ery until 24 h after imbibition (Figure S1), suggesting that Ery does not affect seed germination. Chlorophyll contents in cotyledons were dramatically reduced by Ery (Figure 1C); specifically, both chlorophyll a and chlorophyll b were reduced as the total chlorophyll content was reduced (Figure S2). These combined results indicated that Ery inhibited chlorophyll accumulation, suggesting that Ery may affect photosynthetic metabolic process.

### 3.2. Erythromycin Accumulation in Vegetative Tissue

Ery influences leafy tissue development (Figure 1). We hypothesized that Ery was absorbed through the root and subsequently translocated and accumulated in leaf tissue. To investigate Ery levels in shoots, we collected cotyledons from seedlings grown on agar media in the absence (0 mg/L) and presence of Ery (5 and 10 mg/L) for 4 days and examined Ery levels using LC-MS/MS. Although Ery was applied once by adding to the agar medium, Ery absorption through roots may last until harvesting. The Ery residual levels in seedling aerial parts in the presence of 5 and 10 mg/L Ery were 18.3 ± 1.89 and 34.6 ± 0.99 mg/kg (dried weight), respectively. Three times replicated experiments were exhibited similar value. By contrast, Ery was not detected in seedlings grown in the absence of Ery (0 mg/L). These results revealed that Ery was absorbed from contaminated medium, transported from root to shoot, and accumulated in the shoots. These results suggest that Ery residues accumulate in edible plant tissues and may influence human health.

### 3.3. Differential Proteomic Analysis of Brassica campestris Seedlings

**Figure 2:**
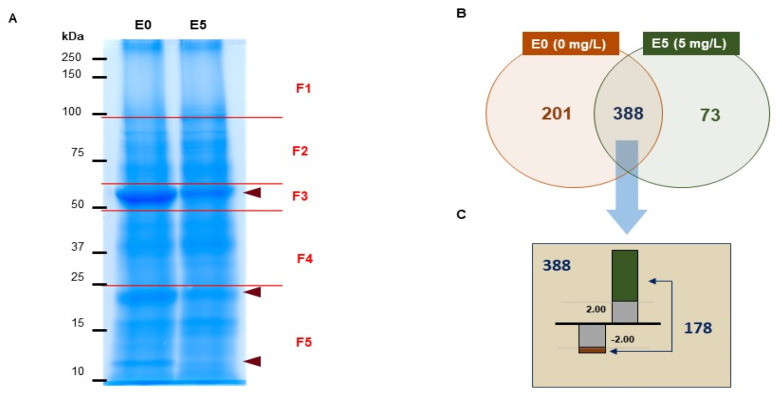
SDS-PAGE image and identified protein levels. (**A**) Coomassie brilliant blue stained gel image. Total proteins were extracted from 4-day-old seedlings grown on either 0 mg/L (E0) or 5 mg/L (E5) Ery and were separated by 12% SDS-PAGE. Separated proteins were divided into five fractions in a size-dependent manner: F1, top~100 kDa; F2, 100~60 kDa; F3, 60~50 kDa; F4, 50~25 kDa; F5, 25 kDa to the bottom. (**B**) Venn diagram of quantified proteins. Proteins were identified by data processing using peptides from LC-MS/MS analyses. (**C**) The number of identified proteins in E0 and E5 samples; 178 proteins of 388 proteins were identified in both E0 and E5 and showed >2-fold difference (false discovery rate (FDR) < 0.01) in abundance.

To better understand the effects of Ery during seedling growth, we performed differentially expressed proteomic analysis using *B. campestris* seedlings grown in the absence (0 mg/L, E0) or presence (5 mg/L, E5) of Ery. First, we separated proteins in a size-dependent manner by subjecting them to 12% SDS-PAGE, observed significantly changed protein abundance between E0 and E5 around 50, 20, and 10 kDa (Figure 2A, arrowhead), and confirmed with four times other biological replicates. As shown by the reduction in chlorophyll contents (Figure 1), those protein bands are predicted as RuBisCO large subunits (rbcL) and RuBisCO small subunits (rbcS) [33]. The most abundant protein in *B. campestris* seedling leaf was RuBisCO, which accounted for 11.56% of total leaf protein [34]. Next, we divided five different fractions (F1 to F5, Figure 2A) and analyzed embedded proteins using LC-MS/MS. In-gel digestion with trypsin and cleanup was performed for loading onto LC-MS/MS and analyzed peptides were quantified and profiled for protein identification (Figure S3). A total of 662 proteins were identified in both E0 and E5 as shown in a Venn diagram analysis (Figure 2B); 73 proteins were specifically expressed in E5 compared to E0, and 201 proteins were not detected in E5. Although 388 proteins were commonly detected in both E0 and E5, >2-fold difference was quantified between E0 and E5 for 178 proteins (FDR < 0.01), with 17 downregulated proteins and 161 upregulated proteins (Figure 2C and Table S2). These results indicated that Ery modulates the levels of many *B. campestris* seedling proteins.

### 3.4. Gene Ontology Analysis of Differentially Expressed Proteins

**Figure 3:**
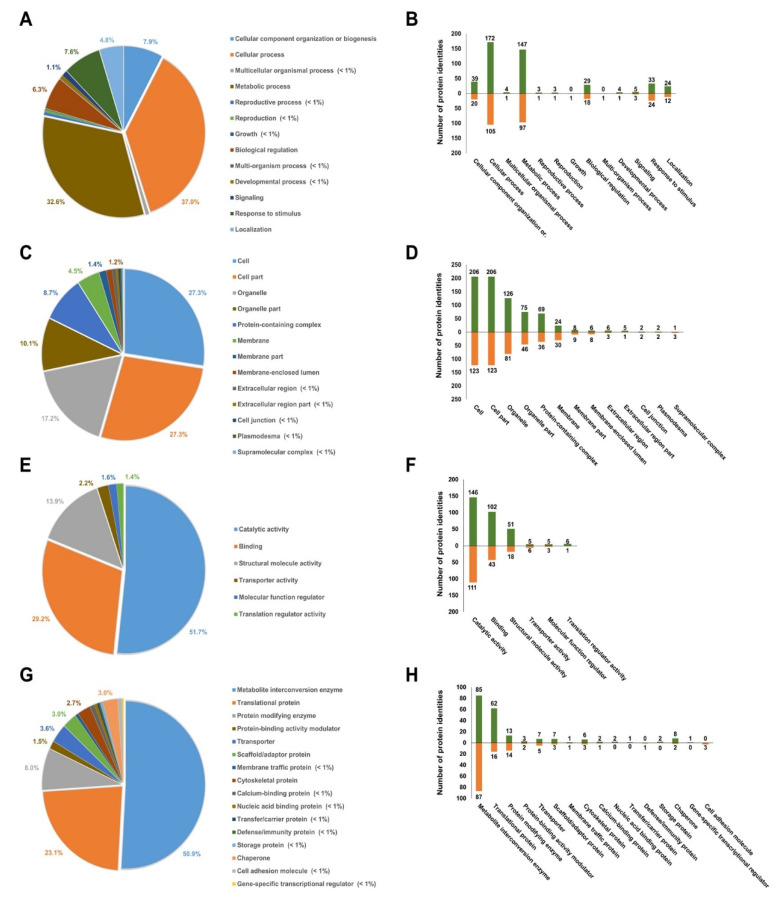
Gene ontology annotation of Ery-responsive proteins. Identified annotated proteins are included and presented according to (**A**) biological process, (**C**) cellular component, (**E**) molecular function, and (**G**) protein class. The percentage distributions of the gene ontology (GO) terms were determined using the PANTHER and STRINGs databases. Protein abundance patterns are presented according to (**B**) biological process, (**D**) molecular function, (**F**) cellular component, and (**H**) protein class. The columns above and under the *x*-axes represent the numbers of upregulated and downregulated proteins by Ery, respectively.

To deduce functional biological processes, the differentially abundant proteins (DAPs) in E5 were analyzed using PANTHER (http://pantherdb.org) and STRING (http://string-db.org) databases and categorized using gene ontology (GO) analysis (Figure S3). GO analysis identified four categories of DAPs: biological process, molecular function, cellular component, and protein class (Figure 3). In the biological process analysis of DAPs, 37% cellular process and 33% metabolic process were dominant among shown other GO biological categories including cellular component organization or biogenesis, localization, biological regulation, response to stimulus, reproductive process, reproduction, multiorganism process, signaling, developmental process, multicellular organismal process, and growth (Figure 3A,B). Proteins involved in reproductive process, signaling, or growth were rarely changed in response to Ery, suggesting that Ery affects energy generation or consumption for plant survival rather than plant reproduction or development. Although most DAPs in the cellular component category were cytosolic proteins with 54% cell and cell part, the second major portion was 27% organelle and organelle parts (Figure 3C,D). The remaining 20% portion was involved in membrane, membrane parts, membrane-enclosed lumen, protein-containing complex, extracellular region, supramolecular complex, cell junction, and plasmodesma. Proteins in the molecular functional level were categorized as follows: 52% catalytic activity, 29% binding, 14% structural molecule activity, and 5% others including transporter and regulator function (Figure 3E). Comparison of E0 and E5 showed that more proteins displayed increased levels than decreased levels (Figure 3F). More specifically, we further classified the protein classes (Figure 3G,H). As expected from biological process and molecular function analysis, 51% metabolic interconversion enzyme and 23% translational proteins represent the major proportions of these classes, with 28% protein modifying enzyme, transporter, scaffold/adaptor protein, chaperone, cell adhesion molecule, protein-binding activity modulator, transfer/carrier protein, nucleic acid binding protein, and cytoskeletal protein. These combined analyses suggested that Ery induced changes in the expression levels of many proteins that may be involved in the function of metabolic enzymes in organelles.

### 3.5. MapMan Pathway Analysis of Differentially Expressed Proteins

**Figure 4:**
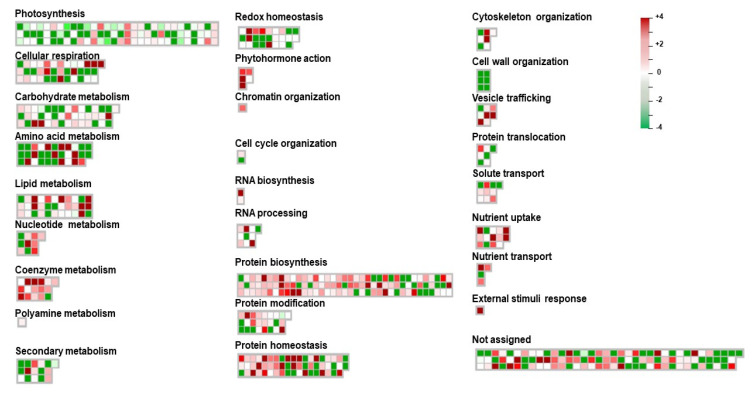
Bin-wise distributions of differentially expressed proteins using MapMan. Relative expression levels (log2) of accumulation are shown by a color gradient from high (red, +4) to low (green, −4).

Our proteomics analysis revealed that Ery modulated the levels of a number of proteins involved in central metabolism. To further identify Ery-induced changes in the functional metabolic pathway during plant growth, we mapped quantified DEP values to MapMan pathways (MapMan ontology version 3.6.0), which refines plant-sourced protein classification and annotation frameworks [32]. Functional pathways contain 18.3% photosynthesis and photorespiration (66 DAPs), 19.4% proteins synthesis (70 DAPs), 21.2% metabolic pathway including carbohydrate metabolism (80 DAPs), 9.4% protein homeostasis (34 DAPs), 3.6% redox homeostasis (13 DAPs), 5% transport (18 DAPs), 2.5% cell structure (9 DAPs), and 19.4% not assigned (70 DAPs) (Figure S4). Although we identified 452 DAPs, 360 DAPs were applied to investigate the protein abundance of individual DAP based on molecular functional category, since 92 DAPs were difficult to use gene accession number, which is required to analyze transcript abundance (Table S2). Then, we analyzed the change in each DAP in the molecular functional pathway to understand the modulation of indicated pathways. Most DAPs in each pathway were distributed with similar numbers of upregulated and downregulated, except for photosynthesis and protein biosynthesis. The photosynthetic pathway and protein biosynthesis pathway had much higher numbers of downregulated DAPs and upregulated DAPs, respectively (Figure S4). The quantified values of characterized proteins (Table S2) were represented in MapMan pathway, and the relative protein abundances were compared in log2 range (Figure 4). As expected, most proteins characterized in the photosynthetic pathway displayed decreased expression levels (color gradient green). By contrast, many protein functions in protein biosynthesis, protein modification, and protein homeostasis displayed increased expression levels (color gradient red) (Figure 4). The list of significantly expressed DAPs in these MapMan pathways are presented in Table 1 for photosynthesis and Table 2 for protein synthesis. Given that all ribosomal proteins are estimated as approximately 8.47% of total lead proteins [34], many ribosomal proteins were identified in Table 2. For the protein annotation in Table 1 and Table 2, we also used Arabidopsis AGI locus as an identifier (http://plants.ensembl.org/Brassica_rapa/; http://brassicadb.org/) due to the lack of *Brassica* gene accessions.

### 3.6. DAPs in Photosynthesis and qRT-PCR Analysis

**Figure 5:**
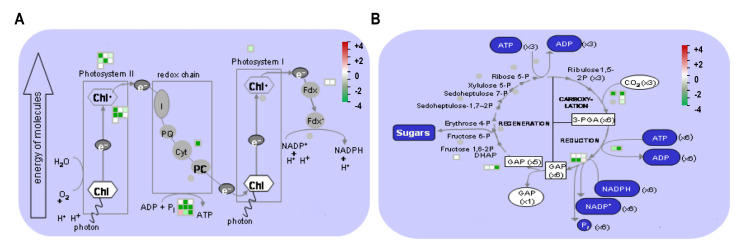
Visualization of molecular processes associated with photosynthesis using MapMan. A photosynthetic pathway showing that 5 mg/L Ery changed protein (colored squares) levels along with log2 values. Color gradients represent the relative differences in protein accumulation from high (red, +4) to low (green, −4). (**A**) Light reactions, thylakoids in the chloroplast (**B**) Calvin cycle, stroma in the chloroplast

Given that Ery downregulated protein function in photosynthesis, we investigated the role of DAPs in the photosynthesis pathway and/or chloroplast function using MapMan (Figure 5). As shown in Table 1, we found that most DAPs (E5) in the photosynthesis pathway had lower quantitative values than expressed proteins in the control (E0) (color gradient green), indicating that Ery reduced the expression levels of protein functions in photosynthesis, including those corresponding to PSII in the light reaction (Figure 5A) and RuBisCO in the Calvin cycle (Figure 5B). This result suggested that Ery reduced photosynthetic activities of these DAPs.

**Figure 6:**
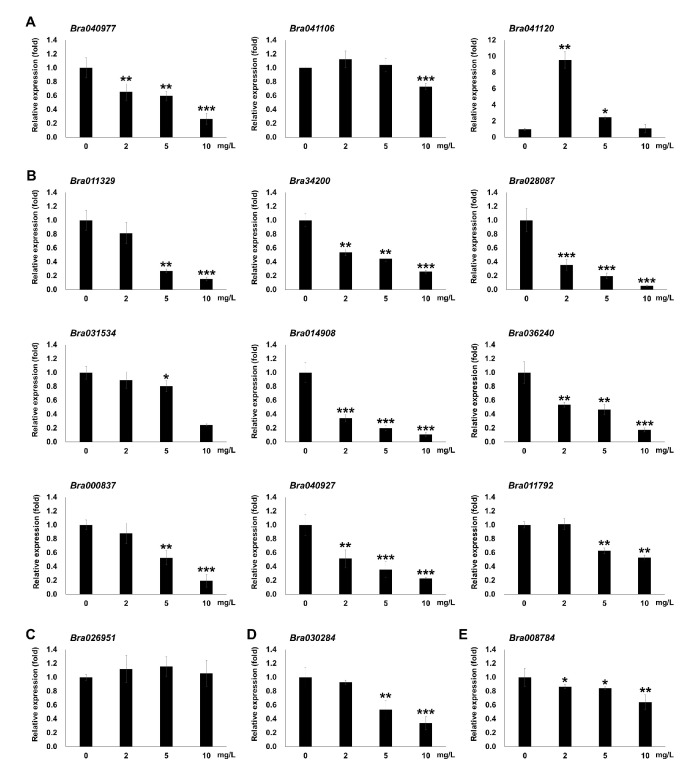
Relative transcript abundance of identified DAPs including the photosynthetic pathway. (**A**) DAPs encoded in chloroplastic genome in the photosynthetic pathway (**B**) DAP genes encoded in nuclear genome in photosynthetic pathway (**C**) Bra026951, a vacuolar ATPase protein (**D**) Bra030284 in the protein synthesis pathway (**E**) Bra008784 from an unassigned group. The relative transcript levels of indicated *B. campestris* genes were analyzed by qRT-PCR. Total RNA was extracted from seedlings grown in the presence of 0, 2, 5, and 10 mg/L Ery for 4 days. Gene expression was normalized to that of EF1a. Bars represent mean ± SD (*n* = 12). The experiments were replicated three times with similar results. Asterisks indicate statistically significant differences relative to the control (0 mg/L Ery). Student’s *t*-test; * *p* < 0.05, ** *p* < 0.01, *** *p* < 0.001.

To further investigate the relationship between DAPs and transcription, we performed qRT-PCR. Although the DAPs were identified from seedlings treated with 5 mg/L Ery (E5), the transcript levels of corresponding proteins were evaluated in seedlings grown on 0, 2, 5, and 10 mg/L Ery (Figure 6). The abundance patterns of targeted *Brassica* genes encoding functional proteins in a photosynthetic pathway (Table S2) were merged with our results in Table 1 and Figure 4 and Figure 6. Photosynthetic-related genes included *Bra040977*, *Bra041106*, *Bra041120*, *Bra011329*, *Bra034200*, *Bra028087*, *Bra031534*, *Bra014908*, *Bra036240*, *Bra000837*, *Bra040927*, *Bra011792*, and *Bra026951*. Some of these genes are encoded in the chloroplastic genome and play roles in the chloroplast according to Arabidopsis identifier information, such as Bra040977, Bra041106, and Bra041120 (Figure 6A). This result indicated that Ery can influence chloroplastic gene expression and chloroplastic protein abundance. Most tested genes in the photosynthetic pathway were downregulated and their gene expression was significantly reduced (Student’s *t*-test; * *p* < 0.05, ** *p* < 0.01, *** *p* < 0.001) (Figure 6B). *Bra026951*, which was annotated as an upregulated DAP, was not induced significantly at the transcriptional level, whereas *Bra041120* was highly upregulated at low Ery concentration (2 mg/L) and dramatically declined at high Ery concentrations (5 and 10 mg/L) (Figure 6A,C). These results suggested that some identified DAP genes were regulated within different temporal points for gene expression or protein expression/stability depending on Ery concentration. We also observed similar consistent results in different pathways; for example, *Bra030284* encoded an RNA-binding protein and was categorized in both protein biosynthesis pathway and RNA processing. *Bra008784* serves a role as a phosphate solute carrier at the mitochondrial membrane in an unassigned group (Figure 6D,E). These results demonstrate that our proteomic and transcript analyses results are consistent.

## 4. Discussion

Environmental residual chemicals such as heavy metals and antimicrobial drugs originating from use in humans and animals affect plant growth and development. These chemicals can accumulate in crop plants, affect food chains, and impact human health [35]. Erythromycin antibiotics have been detected in agricultural fields used for cultivating crops [36,37]. However, it is not well understood how Ery affects crop physiology and/or productivity. This study investigated the effects of Ery in crop growth and development using *B. campestris* seedlings. Although the chemical structure of Ery is not easily absorbed and translocated through cellular membranes [38], Ery accumulation and translocation was reported in hydroponically grown poplar plants irrigated with a recirculating Hoagland‘s nutrient solution containing ~0.01 mg/L Ery [15]. We confirmed Ery accumulation and translocation from root to shoot in *B. campestris*. Poplar plants grown on 1 mg/L Ery exhibited similar health as those grown on 0 mg/L Ery [15], consistent with our observed phenotypes of *B. campestris* grown on 0 and 5 mg/L Ery (Figure 1). These results indicated that low Ery concentrations do not alter morphological phenotypes and suggested that it would be difficult to identify potential toxicity from low Ery concentrations during crop cultivation. By contrast, we observed a severe chlorophyll-deficient phenotype in seedlings grown on higher concentrations than 2 mg/L Ery (Figure 1 and Figure S1) [39], and growth defects at 10 mg/L Ery, suggesting that higher Ery concentrations affect plant growth and development. These combined results suggest that Ery levels detected in the environment (<0.1 mg/L, [36,37]) may not induce morphological and physiological effects and reduced chlorophyll contents due to Ery absorption may not be distinguishable from senescent leaf development during crop cultivation.

Higher Ery concentrations induced physiological differences in *B. campestris* seedlings such as chlorophyll-deficient phenotype (Figure 1 and Figure S1). We identified many Ery-induced differentially abundant proteins (DAPs) (Table S2). These DAPs primarily function in cellular processes and are predicted to have catalytic activity in metabolite interconversion enzyme and protein modifying enzyme (Figure 3). Consistently, studies using a fish report that Ery affects catalytic activities [40,41]. Ery inhibits acetylcholinesterase (AChE) and glutathione S-transferase (GST) activity but enhances superoxide dismutase (SOD) and catalase (CAT) activitys [40,41,42]. Identified DAPs were rarely involved in cell division, cytoskeleton, plant hormone, and vascular tissue development according to classification of biological and molecular functional process, thereby supporting evidence that Ery does not primarily affect morphology.

Ery predominantly inhibits prokaryotic protein synthesis by binding irreversibly to the 50S ribosomal subunit [43,44]. Prokaryotic ribosome is composed of 30S and 50S subunits containing four rRNA species and approximately 55 ribosomal proteins (r-proteins), and its structure and function resemble chloroplast ribosome [45,46]. Functional pathway analysis using MapMan indicated that Ery either directly or indirectly affects photosynthetic protein expression and protein biosynthesis (Figure S4 and Figure 4). Many DAPs in the photosynthetic pathway were downregulated (Figure S4 and Figure 4, Table 1), whereas DAPs classified in protein biosynthesis, modification, and homeostasis were predominantly upregulated (Figure S4 and Figure 4, Table 2). Chloroplast organelles possess an independent protein synthesis pathway [47]. Approximately one-third of chloroplast r-proteins are encoded by the chloroplast genome, and two-thirds are encoded in the nucleus, synthesized in the cytoplasm, and migrate to the chloroplast [48]. As shown in Table 2, Ery contributed to the abundant expression of ribosomal proteins in the chloroplastic genome and nuclear genome compared to the control (E0), suggesting that Ery interrupted the chloroplast translation machinery, including ribosomal structure and organization that results from chloroplastic and nucleic genomes.

Ery affected metabolic process (Figure 3). As photosynthetic activity, which is an important metabolic process in plants [15,42,49], we observed decreases in photosynthetic pigment, chlorophyll content (Figure 1 and Figure S1), a photosynthetic pathway of DAPs functional distribution (Figure 4 and Figure S4), and downregulation of chloroplastic DAPs and/or DAPs functioning in photosynthetic machinery such as rbcL and rbcS (RuBisCO) (Table 1). Chlorophylls play key roles in all aspects of the photosynthetic light reaction, including light harvesting, energy transfer, and light energy conversion, suggesting that the alteration of chlorophyll fluorescence parameters may reflect photosynthesis change. The photosynthetic activity of *Porphyra yezoensis* was reduced by Ery treatment, which reduced F_v_/F_m_ and changed color [49]. Studies using *Microcystis flos-aquae* and *Selenastrum capricornutum* showed that Ery inhibits F_v_/F_m_ and the F_v_/F_0_ ratio by targeting a candidate thylakoid membrane system [42,50]. These Ery-induced effects on chlorophyll fluorescence quenching and F_v_/F_m_ depended on Ery dosage and exposure time [49,51,52]. In the light reaction during photosynthesis, photochemical energy conversion by charge separation in PSII reaction centers is reduced by changes in the linear electron transport rate to PSI through the cytochrome complex [53,54]. As expected, Ery inhibited protein synthesis of components in the thylakoid, including the PSII reaction center and cytochrome complex, and reduces membrane proteins including ATPase (Figure 5A) [50]. These results suggested that Ery inhibited primary photochemistry due to PSII reaction center deficiency and/or disrupted electron transport from PSII to PSI due to cytochrome complex deficiency (Figure 5A) [51]. Studies using eukaryotic green algae that possess chloroplasts showed that levofloxacin and amphotericin B antibiotics significantly inhibit photosynthetic electron transport [55,56].

Ribulose bisphosphate carboxylase is a rate-limiting enzyme in the photosynthetic carbon reduction cycle that catalyzes the first step of the carbon assimilation process (Figure 5B). Ery inhibited the synthesis of ribulose bisphosphate carboxylase subunits (rbcL and rbcS), reduced their contents (Figure 2A, Table S2 and Table 1) [52], interfered with assembly, and attenuated RuBisCO activity of this enzyme. Transgenic tobacco plants expressing <50% of wild-type RuBisCO activity had reduced starch and sucrose levels [57,58], and leaf development and growth of transgenic plants expressing only 20% of normal RuBisCO activity were altered with reduced photosynthetic capacity [59]. Ery also affected photosynthetic gene transcription (Figure 6) [60] and protein abundance (Table 1), suggesting that disrupted electron transport, decreased reducing power of ATP and NADPH levels, and low RuBisCO activity affect carbon fixation and assimilation.

In conclusion, we suggest that environmental Ery acted as a protein synthesis inhibitor, thereby changing protein abundance and modulating plant physiology involved especially in photosynthesis, and crop production. As this is the first proteomics study of the effects of veterinary antibiotics in plants, we also provided a comprehensive overview of proteomics analyses of plant response to macrolide antibiotics. Our study may lead to a broader understanding of molecular and physiological responses to environmental antibiotics in crop plants.

## Figures and Tables

**Table 1:** Significantly Differentially Expressed Proteins by Ery in Photosynthesis (FDR < 0.01).

Protein ID	Molecular Weight	Quantitative Value (Normalized Total Spectra)		*Brassica* Accession	AGI Locus Identifier	Description
		E0	E5			
M4EHZ1	20 kDa	456.8	0.0	*Bra028406*	*AT5G38410*	Ribulose bisphosphate carboxylase small chain
M4EYY5	20 kDa	205.0	0.0	*Bra034027*	*AT1G67090*	Ribulose bisphosphate carboxylase small chain
M4EPE0	28 kDa	81.5	0.0	*Bra030660*	*AT1G06680*	PsbP domain-containing protein
M4DG97	28 kDa	62.4	0.0	*Bra015520*	*AT1G06680*	PsbP domain-containing protein
A0A249RRH7	56 kDa	33.9	0.0	*Bra040977*	*ATCG00680*	Photosystem II CP47 reaction center protein
A0A249RQW1	39 kDa	25.1	0.0		*ATCG00020*	Photosystem II protein D1 (psbA)
M4FBB1	26 kDa	22.4	0.0	*Bra038377*	*AT4G09650*	Uncharacterized protein
M4C9F8	26 kDa	22.4	0.0	*Bra000837*	*AT4G03280*	Plastoquinol–plastocyanin reductase
M4DNQ7	28 kDa	18.3	0.0	*Bra018144*	*AT3G47470*	Chlorophyll a–b binding protein, chloroplastic
M4EV39	23 kDa	15.6	0.0	*Bra032672*	*AT4G12800*	PSI subunit V
A0A249RPW6	9 kDa	15.6	0.0	*Bra041107*	*ATCG00580*	Cytochrome b559 subunit alpha (psbE)
A0A249RQ23	9 kDa	14.3	0.0		*ATCG01060*	Photosystem I iron–sulfur center (psaC)
A0A249RPV2	52 kDa	12.9	0.0	*Bra041123*	*ATCG00280*	Photosystem II CP43 reaction center protein (psbC)
M4DD65	28 kDa	12.2	0.0	*Bra014433*	*AT3G61470*	Chlorophyll a–b binding protein, chloroplastic
M4F5A2	24 kDa	8.8	0.0	*Bra036258*	*AT4G02530*	Uncharacterized protein
M4CLA2	30 kDa	8.1	0.0	*Bra004989*	*AT2G40100*	Chlorophyll a–b binding protein, chloroplastic
A0A249RRE6	40 kDa	6.8	0.0		*ATCG00270*	Photosystem II D2 protein (psbD)
M4D4C5	24 kDa	6.8	0.0	*Bra011329*	*AT4G32260*	Uncharacterized protein
M4CFL7	29 kDa	4.8	0.0	*Bra002999*	*AT5G54270*	Chlorophyll a–b binding protein, chloroplastic
A0A249RPW8	82 kDa	3.4	0.0	*Bra041122*	*ATCG00350*	Photosystem I P700 chlorophyll a apoprotein A2 (psaB)
M4E904 (+2)	32 kDa	2.0	0.0	*Bra025260*	*AT3G27240*	Cytochrome c domain-containing protein
M4C8N9	26 kDa	1.4	0.0	*Bra000567*	*AT3G63540*	PsbP domain-containing protein
M4F584	31 kDa	33.3	3.8	*Bra036240*	*AT4G02770*	Uncharacterized protein
M4EZF7	25 kDa	25.1	5.7	*Bra034200*	*AT4G03280*	Plastoquinol–plastocyanin reductase
M4C909	25 kDa	29.9	7.6	*Bra000687*	*AT4G09650*	Uncharacterized protein
M4ELR3	31 kDa	26.5	11.4	*Bra029732*	*AT3G08940*	Chlorophyll a–b binding protein, chloroplastic
M4DEI7	24 kDa	38.7	13.3	*Bra014908*	*AT1G31330*	PSI-F
M4E725	53 kDa	48.2	17.1	*Bra024580*	*AT1G23310*	Aminotran_1_2 domain-containing protein
M4FIJ8	16 kDa	5.4	19.0	*Bra040927*	*AT2G28900*	Uncharacterized protein
M4EDU1	65 kDa	4.1	19.0	*Bra026951*	*AT1G12840*	V-type proton ATPase subunit C
A0A249RPX1	35 kDa	59.7	26.6	*Bra041106*	*ATCG00540*	Cytochrome f (petA)
M4ERV7	28 kDa	82.1	38.0	*Bra031534*	*AT1G06680*	PsbP domain-containing protein
M4D5N5	73 kDa	26.5	53.2	*Bra011792*	*AT4G37870*	Phosphoenolpyruvate carboxykinase (ATP)
A0A249RRD6	55 kDa	292.5	146.2	*Bra041120*	*ATCG00120*	ATP synthase subunit alpha, chloroplastic
M4EH22	53 kDa	2700.1	1086.0	*Bra028087*	*ATCG00490*	Ribulose bisphosphate carboxylase large chain (rbcL)

**Table 2:** Significantly Differentially Expressed Proteins by Ery in Protein Synthesis (FDR < 0.01).

Protein ID	Molecular Weight	Quantitative Value (Normalized Total Spectra)		*Brassica* Accession	AGI Locus Identifier	Description
		E0	E5			
M4D1Y3	32 kDa	9.5	0.0	*Bra010483*	*AT5G50250*	Uncharacterized protein
M4CNF7	15 kDa	8.8	0.0	*Bra005745*	*AT5G02960*	40S ribosomal protein S23
M4EQE3	36 kDa	8.8	0.0	*Bra031014*	*AT1G18080*	WD_REPEATS_REGION domain-containing protein
M4D7B0	17 kDa	7.5	0.0	*Bra012370*	*AT1G23290*	Ribosomal_L18e/L15P domain-containing protein
M4DFM6	17 kDa	7.5	0.0	*Bra015299*	*AT1G04270*	Structural constituent of ribosome
M4CHU3	16 kDa	6.8	0.0	*Bra003776*	*AT1G74970*	Structural constituent of ribosome
M4DZD2	32 kDa	6.8	0.0	*Bra021879*	*AT2G33800*	S5 DRBM domain-containing protein
M4E880	45 kDa	6.8	0.0	*Bra024986*	*AT1G43170*	Structural constituent of ribosome
A0A249RQ06	11 kDa	6.8	0.0	*Bra001922*	*AT5G47320*	30S ribosomal protein S19, chloroplastic (rps19)
A0A249RPV7	23 kDa	6.8	0.0	*Bra027599*	*AT5G45250*	30S ribosomal protein S4, chloroplastic (rps4)
M4D384	26 kDa	4.8	0.0	*Bra010937*	*AT1G27450*	Adenine phosphoribosyltransferase
M4C7V7	29 kDa	4.1	0.0	*Bra000285*	*AT2G43030*	Ribosomal protein L3 family protein
M4DCB8	19 kDa	4.1	0.0	*Bra014131*	*AT1G48350*	Ribosomal protein L18 family protein
M4DJ77	26 kDa	4.1	0.0	*Bra016555*	*AT1G18540*	Ribosomal_L6e_N domain-containing protein
M4CPV9	19 kDa	3.4	0.0	*Bra006248*	*AT5G14320*	30S ribosomal protein S13, chloroplast
A0A249RPV9	27 kDa	3.4	0.0	*Bra013947*	*AT4G26090*	30S ribosomal protein S2, chloroplastic (rps2)
A0A249RR23	30 kDa	3.4	0.0		*ATCG00830*	50S ribosomal protein L2, chloroplastic (rpl2)
M4DDG7	13 kDa	2.7	0.0	*Bra014535*	*AT2G43460*	60S ribosomal protein L38
M4F6Q3	22 kDa	2.7	0.0	*Bra036763*	*AT1G35680*	50S ribosomal protein L21, chloroplastic
M4CX77	24 kDa	2.0	0.0	*Bra008824*	*AT5G13510*	Ribosomal protein L10 family protein
A0A249RRI7	14 kDa	2.0	0.0	*Bra040980*	*ATCG00780*	50S ribosomal protein L14, chloroplastic (rpl14)
M4DXP8	36 kDa	1.4	0.0	*Bra021294*	*AT3G18130*	WD_REPEATS_REGION domain-containing protein
A0A249RQJ6	18 kDa	1.4	0.0		*ATCG00810*	50S ribosomal protein L22, chloroplastic (rpl22)
M4ENY9	25 kDa	0.0	3.8	*Bra030509*	*AT1G02780*	Ribosomal protein L19
M4EZX0	11 kDa	1.4	5.7	*Bra034363*	*AT2G27710*	60S acidic ribosomal protein P2
M4D4S2	6 kDa	0.0	5.7	*Bra011477*	*AT3G44010*	40S ribosomal protein S29
M4CVX6	27 kDa	2.0	7.6	*Bra008373*	*AT1G78630*	Structural constituent of ribosome
M4D857	24 kDa	3.4	9.5	*Bra012667*	*AT4G16720*	Ribosomal protein L15
M4DLG0	30 kDa	3.4	9.5	*Bra017341*	*AT3G25920*	Ribosomal_L18e/L15P domain-containing protein
M4CCC7	16 kDa	0.0	9.5	*Bra001857*	*AT4G15000*	60S ribosomal protein L27
M4CHH9	12 kDa	4.8	11.4	*Bra003662*	*AT1G77940*	Ribosomal_L7Ae domain-containing protein
M4EMA8	18 kDa	5.4	13.3	*Bra029928*	*AT5G23740*	Ribosomal_S17_N domain-containing protein
M4FD44	16 kDa	0.0	13.3	*Bra039014*	*AT2G19730*	Ribosomal_L28e domain-containing protein
M4CIF0	21 kDa	7.5	15.2	*Bra003984*	*AT1G66580*	Ribosomal_L16 domain-containing protein
M4DVY7	24 kDa	6.8	15.2	*Bra020681*	*AT5G48760*	60S ribosomal protein L13A
M4C811	84 kDa	5.4	15.2	*Bra000339*	*AT2G44060*	6,7-dimethyl-8-ribityllumazine synthase
M4D8V6	17 kDa	3.4	15.2	*Bra012916*	*AT3G49910*	KOW domain-containing protein
M4DU07	19 kDa	3.4	15.2	*Bra020000*	*AT1G09590*	60S ribosomal protein L21
M4DW28	10 kDa	3.4	15.2	*Bra020722*	*AT3G61110*	40S ribosomal protein S27
M4EE11	85 kDa	0.0	15.2	*Bra027021*	*AT1G62750*	Elongation factor G, chloroplastic
M4E3F1	23 kDa	2.7	17.1	*Bra023302*	*AT1G32990*	Structural constituent of ribosome
M4DQ40	25 kDa	0.0	17.1	*Bra018631*	*AT2G27530*	Ribosomal protein
M4DWX7	16 kDa	8.8	19.0	*Bra012616*	*AT4G18100*	60S ribosomal protein L32
M4DVR1	47 kDa	8.8	19.0	*Bra020605*	*AT5G28020*	Cysteine synthase
M4CUN4	16 kDa	6.8	19.0	*Bra007929*	*AT1G70600*	Ribosomal_L18e/L15P domain-containing protein
M4CFK1	13 kDa	4.8	19.0	*Bra002983*	*AT5G04800*	40S ribosomal protein S17
M4EBR8	73 kDa	10.2	22.8	*Bra026227*	*AT1G30580*	Obg-like ATPase 1
M4DND0	24 kDa	9.5	22.8	*Bra018017*	*AT3G49010*	60S ribosomal protein L13
M4CAC5	14 kDa	12.2	24.7	*Bra001154*	*AT3G05560*	60S ribosomal protein L22-2
M4D389	23 kDa	12.2	24.7	*Bra010943*	*AT1G27400*	60S ribosomal protein L17
M4CQ00	23 kDa	8.8	24.7	*Bra006289*	*AT5G15200*	S4 RNA-binding domain-containing protein
M4FGB7	17 kDa	8.1	28.5	*Bra040145*	*AT5G18380*	40S ribosomal protein S16
M4C7F7	17 kDa	5.4	28.5	*Bra000135*	*AT2G39460*	Ribosomal_L23eN domain-containing protein
M4D1R2	16 kDa	8.1	30.4	*Bra010412*	*AT4G27090*	Ribosomal_L14e domain-containing protein
M4EBD3	13 kDa	7.5	30.4	*Bra026092*	*AT2G32060*	40S ribosomal protein S12
M4FH70	37 kDa	13.6	32.3	*Bra040448*	*AT3G63490*	Ribosomal protein
M4D5W4	12 kDa	12.9	32.3	*Bra010696*	*AT4G39200*	40S ribosomal protein S25
M4CGU4	17 kDa	17.0	36.1	*Bra003427*	*AT4G00100*	Ribosomal_S13_N domain-containing protein
M4D7X0	21 kDa	15.6	38.0	*Bra012580*	*AT4G18730*	Structural constituent of ribosome
M4EHJ8	14 kDa	10.9	38.0	*Bra028263*	*AT5G62300*	Ribosomal_S10 domain-containing protein
M4FGG9	105 kDa	10.9	39.9	*Bra040197*	*AT3G04380*	Histone-lysine N-methyltransferase
P51423	15 kDa	17.6	41.8	*Bra029570*	*AT4G02890*	Ubiquitin-60S ribosomal protein L40 (RL40_BRARP)
M4DL77	37 kDa	12.2	41.8	*Bra017258*	*AT2G36145*	Uncharacterized protein
M4CS06	18 kDa	20.4	45.6	*Bra006998*	*AT3G53430*	60S ribosomal protein L12
M4DIF5	17 kDa	12.2	53.2	*Bra016282*	*AT1G26630*	Eukaryotic translation initiation factor 5A
M4D178	28 kDa	21.0	57.0	*Bra010227*	*AT4G31700*	40S ribosomal protein S6
M4EGW3	25 kDa	27.8	62.7	*Bra028028*	*AT1G34030*	40S ribosomal protein S18
M4EZ44	38 kDa	30.5	64.6	*Bra034087*	*AT3G09820*	Adenosine kinase
M4CUY5	32 kDa	29.2	64.6	*Bra008030*	*AT1G72370*	40S ribosomal protein SA
M4DLT0	28 kDa	31.2	77.8	*Bra017461*	*AT2G01250*	60S ribosomal protein L7
M4ELW0	45 kDa	34.6	79.7	*Bra029780*	*AT3G09630*	Ribos_L4_asso_C domain-containing protein
M4CEM1	236 kDa	30.5	85.4	*Bra002652*	*AT5G58410*	RING-type E3 ubiquitin transferase
M4D611	65 kDa	48.2	163.3	*Bra011919*	*AT1G55490*	ATP binding/protein binding
M4EPY7	74 kDa	46.8	172.8	*Bra030858*	*AT1G55490*	ATP binding/protein binding

## Supplementary Materials

The following are available online at https://www.mdpi.com/2075-1729/10/12/311/s1. Figure S1. Ery effect on seed germination; Figure S2. Chlorophyll a and chlorophyll b levels with and without Ery treatment on *B. campestris* seedlings; Figure S3. Schematic chart of proteomic workflow; Figure S4. Protein abundance using functional categories; Supplementary Table S1. Primers used in qRT-PCR analyses; Supplementary Table S2. List of identified differentially expressed proteins modulated by Ery (FDR < 0.01).
