# Supplementary material for: Erythromycin Treatment of Brassica campestris Seedlings Impacts the Photosynthetic and Protein Synthesis Pathways
Source: Life (Basel). 2020 Nov 26;10(12):311. doi: 10.3390/life10120311 (PMC7759809; doi:10.3390/life10120311)
Supplement: Supplementary file 1 [file life-10-00311-s001.zip › Supplementary file.docx]

Supporting information

Proteomic analysis of erythromycin-induced growth changes in *Brassica campestris* seedlings

Young-Eun Yoon ^2,¶^, Hyun Min Cho ^2,¶^, Dong-won Bae ^3^, Sung Joong Lee^3^, Hyeonji Choe^2^, Min Chul Kim ^1,2^ , Mi Sun Cheong ^1,2^ *, and Yong Bok Lee^1,^ ^2,^*

^1^ Institute of Agriculture & Life Science, Gyeongsang National University, Jinju, Korea

^2^ Division of Applied Life Science (BK21four), Gyeongsang National University, Jinju, Korea

^3^ Center for Research Facilities, Gyeongsang National University, Jinju, Korea

^¶^ These authors contributed equally in this study

***** Co-correspondence: mscheong@gnu.ac.kr; Tel.: +82-55-772-1967 (M.S.C)

***** Correspondence: yblee@gnu.ac.kr; Tel.: +82-55-772-1967 (Y. B. L)

**Supplementary Materials:** The following are available online at www.mdpi.com/xxx/s1.

**Figure S1.** Ery effect on seed germination.

**Figure S2.** Chlorophyll a and chlorophyll b levels with and without Ery treatment on *B.* *campestris* seedlings.

**Figure S3.** Schematic chart of proteomic workflow.

**Figure S4.** Protein abundance using functional categories.

**Supplementary Table S1.** Primers used in qRT-PCR analyses.

**Supplementary Table S2.** List of identified differentially expressed by Ery (FDR<0.01).

**Supplementary Table S1.** Primers used in qRT-PCR analyses.

| **Gene** | **Primer Name** | **Sequence (5’→3’)** | |  |
| --- | --- | --- | --- | --- |
| *EF1a* | EF1a-qRT-F | CCCTCCGTCTACCACTTCAG | | Normalization |
|  | EF1a-qRT-F | CACAACCATACCAGGCTTGA | |  |
| *ACT7* | ACT7-qRT-F | CTACGAGTTACCTGATGGA | |  |
|  | ACT7-qRT-R | ATGATGGAGTTGTAAGTTGTC | |  |
| *Bra040977* | Bra040977-qRT-F | CCGGGCTACTTTGAAATCTG | |  |
|  | Bra040977-qRT-R | TTGGATCTCCCACTTTTTGG | |  |
| *Bra041106* | Bra041106-qRT-F | TGGACGCCAAGTGATTGATA | |  |
|  | Bra041106-qRT-R | AAGGCCTTGGACACGTAATG | |  |
| *Bra041120* | Bra041120-qRT-F | AGCTGGCAAATCAAAATTGG |  | |
|  | Bra041120-qRT-R | TTCCACTGGGAGAGGATTTG |  | |
| *Bra026951* | Bra026951-qRT-F | ACACAGCGTTGAAGCACAAG |  | |
|  | Bra026951-qRT-R | TCAGCGAATATACGCACAGC |  | |
| *Bra029732* | Bra029732-qRT-F | GGTTTCCGACATTAGCTCCA |  | |
|  | Bra029732-qRT-R | TGCCTAAACCAAAAGGATCG |  | |
| *Bra025260* | Bra025260-qRT-F | AATTGCTATGCCGAAAATGC |  | |
|  | Bra025260-qRT-R | GCAGAGCCAGCGATAGTAGG |  | |
| *Bra011329* | Bra011329-qRT-F | CCCTCGGGAACTTTATGGAT |  | |
|  | Bra011329-qRT-R | CCACCTTCTTCCTCCCTACC |  | |
| *Bra034200* | Bra034200-qRT-F | ATGCCCTTGGAAACGATGTA |  | |
|  | Bra034200-qRT-R | TGGCACAACACATCCAAGAT |  | |
| *Bra028087* | Bra028087-qRT-F | TTGCCGAGATAATGGCCTAC |  | |
|  | Bra028087-qRT-R | TCATCGCGCAGTAAATCAAC |  | |
| *Bra031534* | Bra031534-qRT-F | GATGGGTTCAAAGTGCAGGT |  | |
|  | Bra031534-qRT-R | TTCGGGAGAACCGTAATCAG |  | |
| *Bra014908* | Bra014908-qRT-F | GACGGTTTACCGCACTTGAT |  | |
|  | Bra014908-qRT-R | CGGAAGATGATCCGACTAGC |  | |
| *Bra036240* | Bra036240-qRT-F | GAAGGTCCGAACTTGCTGAA |  | |
|  | Bra036240-qRT-R | CTCCGGATAAACCCCATCTT |  | |
| *Bra000837* | Bra000837-qRT-F | CTACCTTCTTTGCCCCTC CT |  | |
|  | Bra000837-qRT-R | ATGGCACAACACATCCAAGA |  | |
|  |  |  |  | |
| *Bra040927* | Bra040927-qRT-F | CCATCGACAAAGGGAGTGTT |  | |
|  | Bra040927-qRT-R | AGCATTGCGTTTTTCCAATC |  | |
| *Bra011792* | Bra011792-qRT-F | GAGAACACACGTGCTGCCTA |  | |
|  | Bra011792-qRT-R | CCAGCAACCAGAGCAGTGTA |  | |


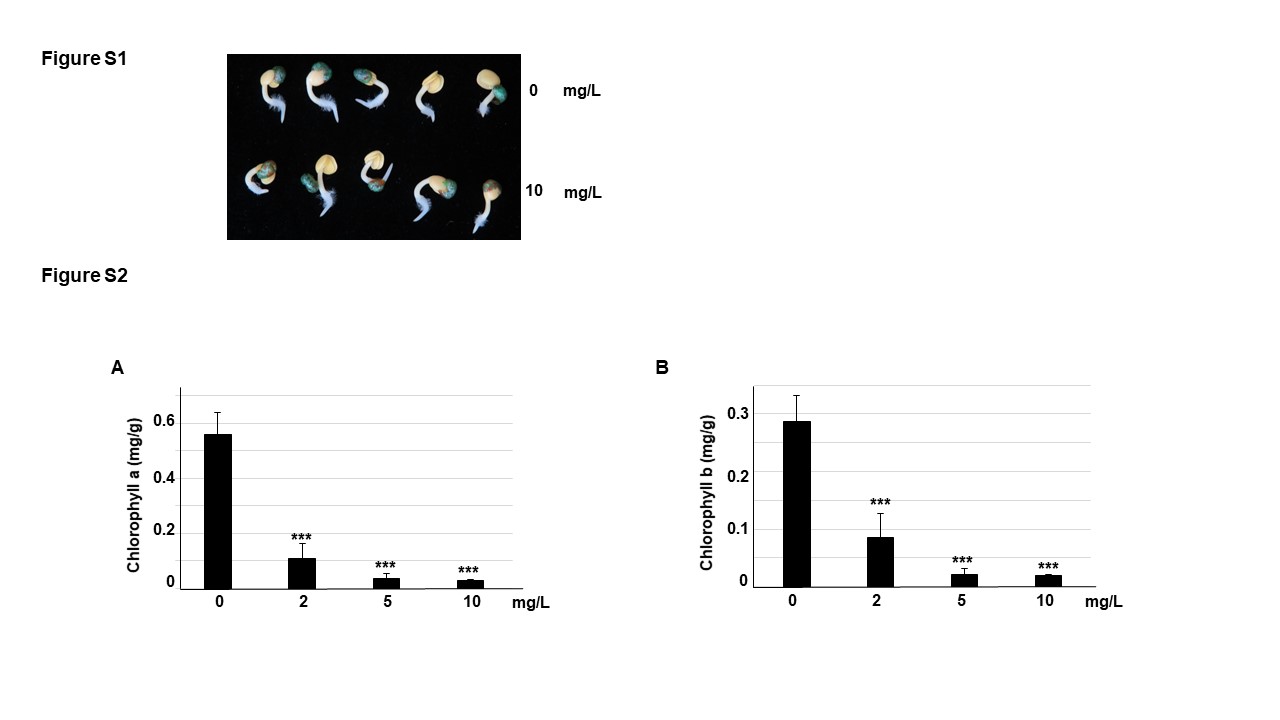


**Figure S1.** Erythromycin effect on seed germination. Sterilized seeds were cultured in deionized distilled water in the presence of 0 or 10 mg/L Ery. A photograph was taken at 24 hours post-imbibition. Experiments were repeated independently three times with similar results.


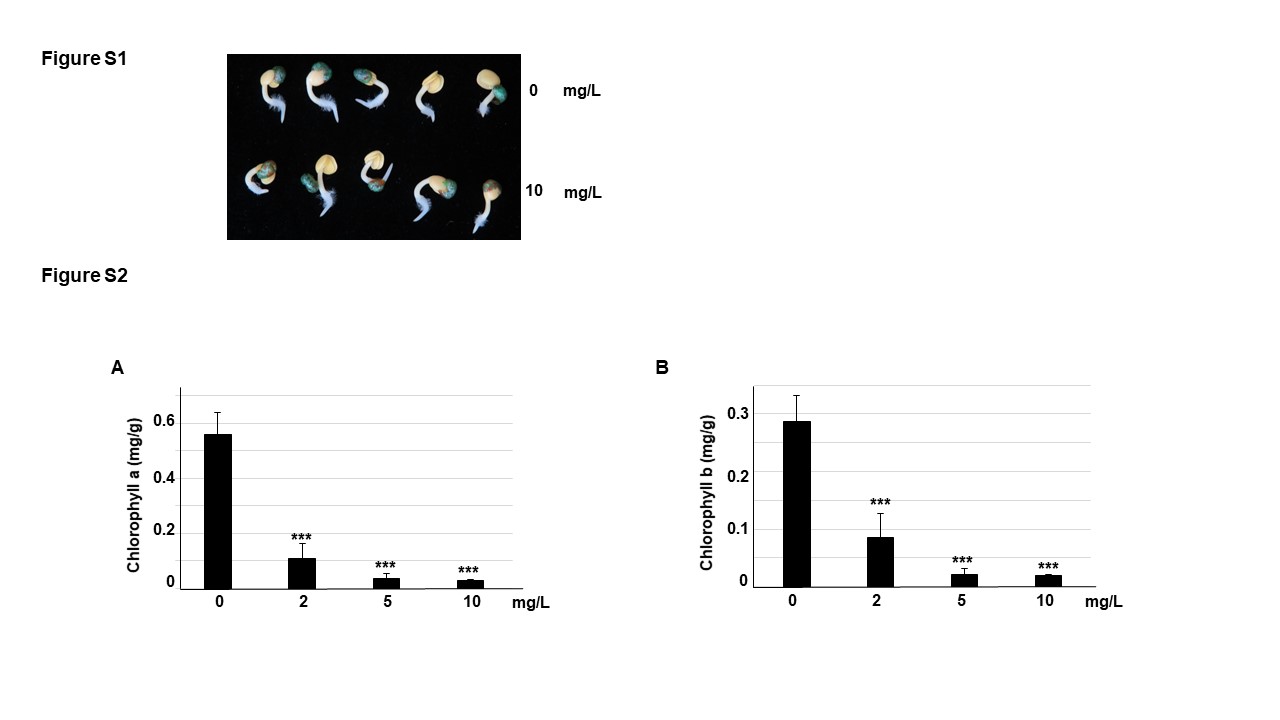


**Figure S2.** Chlorophyll a and chlorophyll b levels with and without Ery treatment on *B.* *campestris* seedlings. Chlorophyll was extracted from cotyledons of 4-day-old *B.* *campestris* seedlings using methanol. (**A**) Chlorophyll a. (**B**) Chlorophyll b. Independent experiments were repeated four times with similar results. The values represent mean ± SD (*n*=16). Asterisks indicate statistically significant difference from 0 mg/L (Student’s *t*-test; **p*<0.05, ***p*<0.01, ****p*<0.001).


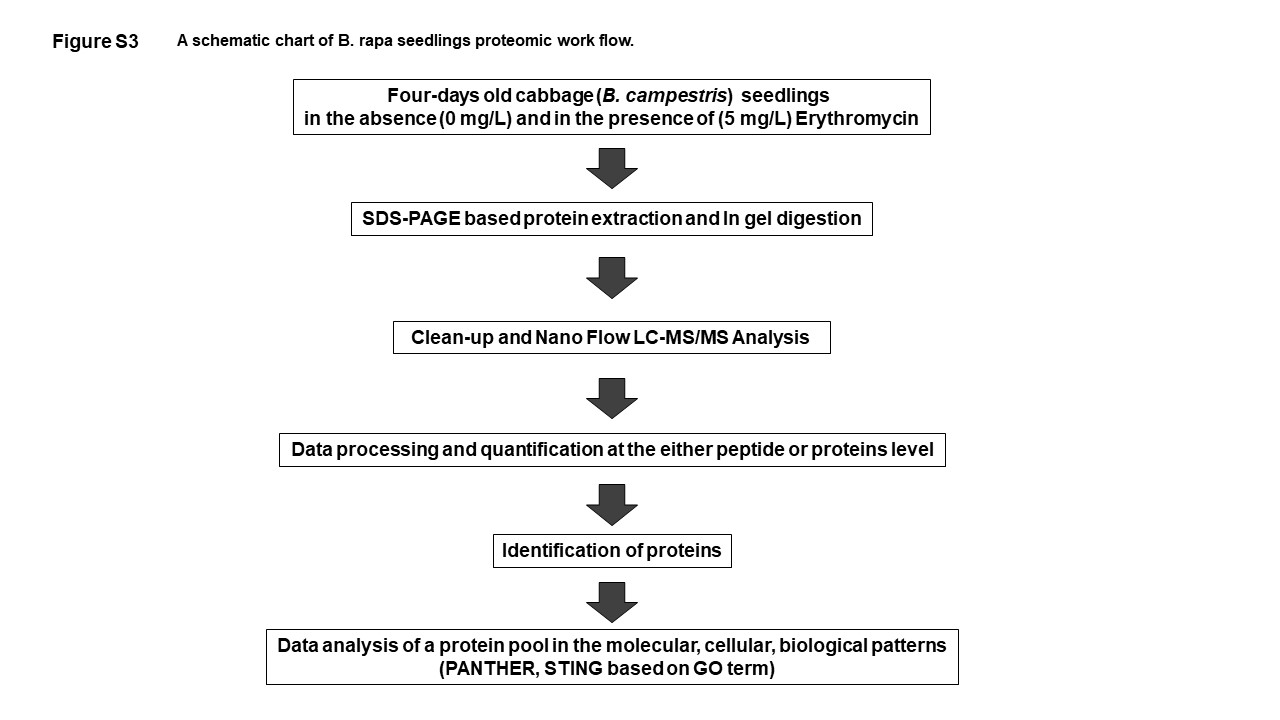


**Figure S3.** Schematic chart of proteomic workflow of high-throughput profiling with one-dimensional analysis. The workflow highlights four key steps from sample preparation to gene ontology categorization. These steps include protein extraction, in-gel digestion with trypsin, clean-up for LC-MS/MS analysis, and quantification of peptide and/or protein levels.


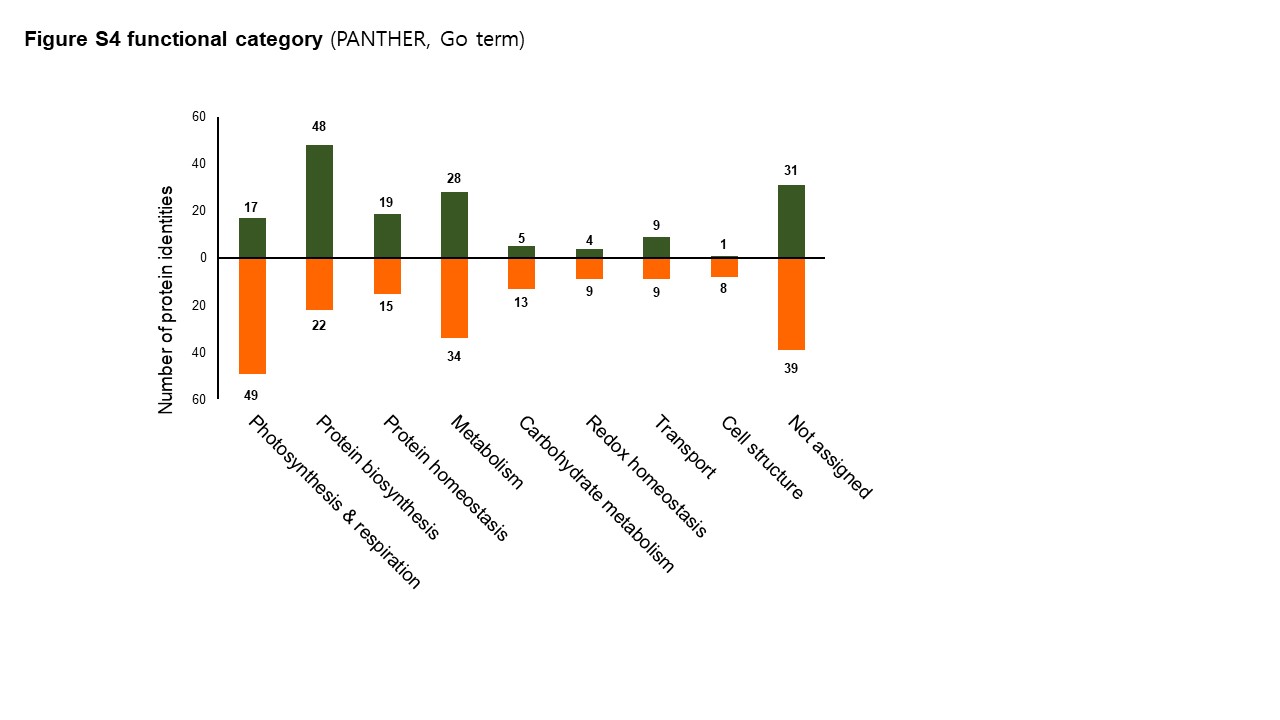


**Figure S4.** Protein abundance using functional categories. The columns above and below the *x*-axis represent the numbers of upregulated and downregulated proteins, respectively, in response to Ery in each functional category.
